# Melanoma Intelligence: Explainable AI Reveals Histopathologic Aggressiveness as the Dominant Axis of Lymph-Node Metastasis

**DOI:** 10.3390/jcm15186945

**Published:** 2026-09-08

**Authors:** Vlad-Petre Atanasescu, Valentin Titus Grigorean, Raluca Florentina Tulin, Maria Fulina, Matei Șerban, Răzvan-Adrian Covache-Busuioc, Corneliu Toader, Alexandru Vlad Ciurea, Anamaria Oproiu

**Affiliations:** 1 Doctoral School, “Carol Davila” University of Medicine and Pharmacy, 050474 Bucharest, Romania; 2 Department of Plastic Surgery, “Carol Davila” University of Medicine and Pharmacy, 050474 Bucharest, Romania; 3 Faculty of General Medicine, “Carol Davila” University of Medicine and Pharmacy, 050474 Bucharest, Romania; mateiserban@innbn.com (M.Ș.);; 4 Department of Anatomy, “Carol Davila” University of Medicine and Pharmacy, 050474 Bucharest, Romania; 5 County Clinical Emergency Hospital “Sf. Ap. Andrei”, 900591 Constanta, Romania; maria.fulina@yahoo.com; 6 Department of Neurosurgery, “Carol Davila” University of Medicine and Pharmacy, 050474 Bucharest, Romania; 7 Department of Vascular Neurosurgery, National Institute of Neurology and Neurovascular Diseases, 077160 Bucharest, Romania; 8 Puls Med Association, 051885 Bucharest, Romania; 9 Medical Section, Romanian Academy, 010071 Bucharest, Romania

**Keywords:** malignant melanoma, lymph-node metastasis, histopathology, Breslow thickness, lymphovascular invasion, explainable artificial intelligence, machine learning, inflammatory–metabolic dysregulation, digital twin, conformal prediction, melanoma risk modeling

## Abstract

**Background/Objectives:** Lymph-node metastasis remains central to staging, prognosis, surveillance, and treatment planning in malignant melanoma. At present, most statistical models assessing nodal metastatic risk in malignant melanoma consider pathological descriptors, inflammatory markers, metabolic alterations, clinical data, and related variables independently of each other. Therefore, we developed a transparent artificial intelligence (AI)-based approach to assess whether the propensity for nodal metastasis is determined by a single layer of local histopathological aggressiveness or by the integration of different biological levels, including local histopathological aggressiveness, systemic inflammatory–metabolic dysregulation, biological heterogeneity, or a clinicobiological pattern. **Methods:** In this retrospective study, we assessed 73 adult patients undergoing surgical removal of malignant melanoma. The primary endpoint was histopathologically confirmed lymph-node metastasis. Routinely collected patient-related data, including clinical, anatomical, operative, histopathological, nodal, comorbidity, biological, clinical course, and available staging data, were structured into interpretable constructs. These included the Histopathologic Aggressiveness Index (HAI), the Inflammatory–Metabolic Dysregulation Index (IMDI), the Biological–Histological Discordance Score (BHDS), model-estimated nodal metastatic probability, integrated clinicobiological risk, and explanation stability. The AI-based framework was evaluated by applying bias-reduced and penalized logistic regression, machine learning benchmarking, leave-one-out cross-validation, bootstrap estimation, permutation testing, decision curve analysis, rule extraction, feature stability evaluation, network analysis, similarity-based retrieval, conformal uncertainty estimation, and unsupervised phenomapping. **Results:** For 72 out of 73 patients, nodal histopathology results were available. Among these patients, 22 had positive nodal status. Positive nodal status was associated with a higher Breslow thickness, an increased mitotic rate, ulceration, lymphovascular invasion, a nodular subtype, and palpable adenopathy. The HAI demonstrated the strongest discriminative signal between node-positive and node-negative patients (median values of 67.8 vs. 45.3; *p* < 0.001) and retained an independent association with nodal metastasis within the bias-reduced logistic model (odds ratio [OR] per 10-point increase: 2.74; 95% confidence interval [CI]: 1.58–4.75; *p* < 0.001). The IMDI showed a weak exploratory relationship and did not retain an independent association after adjustment. Similarly, the BHDS did not show significant differences in separating the two endpoint groups. The penalized logistic model including only the HAI showed good performance under leave-one-out cross-validation, with ROC AUC = 0.889, PR-AUC = 0.706, and Brier score = 0.137. Through rule extraction, we found a cohort-specific HAI threshold value > 58.6, above which all node-positive cases were located. With respect to explainability, feature stability, network analysis, similarity retrieval, conformal prediction, and phenomapping, there was convergence toward a dominant high-risk phenotype defined primarily by histopathological criteria. **Conclusions:** Routine melanoma registries may be transformed into internally evaluated melanoma intelligence frameworks. Histopathologically confirmed lymph-node metastasis among patients with malignant melanoma was organized primarily along an axis of local histopathological aggressiveness, while systemic inflammatory–metabolic dysregulation provided subordinate contextual biological information.

## 1. Introduction

Melanoma progression involves both local tumor behavior and the overall biological status of the host. While many traditional pathological factors, including Breslow thickness, ulceration, mitotic rate, lymphovascular invasion, growth pattern, and melanoma subtype, provide critical information regarding the risk of regional dissemination through their association with different aspects of invasive potential, proliferative activity, tissue destruction, and access to the lymphatic circulation, they represent separate but interrelated biological processes [1]. Therefore, an important question arises as to whether the combination of these different pieces of information can provide a comprehensive representation of the pathological state associated with melanoma progression, or simply a number of highly correlated predictors [2].

Another level at which to view the disease process is through the lens of inflammatory–metabolic biology. Several measures that have been used to assess the degree of inflammation and/or metabolic dysregulation in patients with cancer include C-reactive protein, interleukin-6, lactate dehydrogenase, glucose, vitamin D, and calcium-related physiology [3]. Many of these measures are easily obtained and widely available; however, each is relatively nonspecific and subject to variability based on multiple factors, including timing, the presence of other diseases, nutritional status, and other physiological influences [4]. Therefore, it is reasonable to assess their value for predicting regional spread of melanoma not only on the basis of their individual associations with the disease process, but also by determining whether they add useful information to that provided by the local pathology of the primary tumor [5].

Given the reality of melanoma registries, these considerations take on increased importance. Frequently, registries contain large numbers of correlated pathological variables alongside incomplete or missing biomarkers, varying degrees of completeness in staging information, and little or no molecular characterization [6]. As a result, adding layers of complexity to models of melanoma risk does not necessarily lead to greater biological understanding and may instead produce unstable results. Under these conditions, the more meaningful problem may be determining whether heterogeneous routine data can be placed within identifiable biological frameworks that allow us to understand how much predictive information they contain and what types of uncertainty surround them [7].

The conceptual novelty of our approach lies in treating melanoma modeling as a problem of structuring biological information in order to compress it, rather than simply selecting algorithms. We, therefore, sought to determine whether multidimensional histopathological data could be compressed into a single interpretable dimension representing aggressiveness while retaining clinically relevant information; whether this dimension retained information beyond that found when examining its strongest individual component; and whether systemic inflammatory–metabolic dysregulation contributed independently to the prediction of nodal metastasis or provided an alternative way of viewing clinicobiological differences among patients.

To examine these questions, locally aggressive tumor characteristics were represented using a Histopathologic Aggressiveness Index (HAI); systemic dysregulation was represented using an Inflammatory–Metabolic Dysregulation Index (IMDI); and the relationship between these two domains was assessed using a Biological–Histological Discordance Score (BHDS). These constructs were designed to represent complementary biological domains and their relationship rather than to replace their individual components or existing staging systems.

Accordingly, this proof-of-concept study developed and internally evaluated an analytically based framework for assessing histopathologically confirmed lymph-node metastasis in patients with melanoma using routinely collected clinicopathological and inflammatory–metabolic information. Specifically, we investigated whether the propensity for nodal metastasis was organized primarily along a multidimensional axis of local histopathological aggressiveness; whether systemic dysregulation contributed predictive information beyond that provided by local histopathology; and whether discordance between these two domains identified additional clinicobiological phenotypes.

We employed established statistical and machine learning methods as complementary analytical approaches to test whether the same underlying biological organization remained evident across predictive performance, quantitative explanation of model predictions, resampling stability, uncertainty estimation, cross-domain relationships, and clustering of patient-level characteristics. Our goal was to develop a hypothesis-generating research architecture rather than a deployable clinical decision support tool for melanoma risk assessment. Independent validation will, therefore, be required before any resulting score, phenotype, or decision rule can be considered for clinical application.

## 2. Materials and Methods

This single-center, retrospective, proof-of-concept study utilized a malignant melanoma registry containing all relevant variables of interest. The patient enrollment period extended from February 2024 to May 2026. The primary objective of this investigation was to evaluate whether histopathologically confirmed lymph-node metastasis (LNM) occurred predominantly along a local histopathological aggressiveness axis or whether other potentially predictive or phenotypic information could be generated from systemic inflammatory/metabolic biology and/or from the relationship between these two biological domains.

Instead of loading all of the routinely collected variables into a single high-dimensional model, the analytical structure defined three layers of biologically coherent variables. The first layer, representing the degree of local tumor aggressiveness, was represented by the Histopathologic Aggressiveness Index (HAI). The second layer, representing the degree of systemic inflammatory/metabolic dysregulation, was represented by the Inflammatory–Metabolic Dysregulation Index (IMDI). Finally, the third layer, representing the extent of discrepancy between these two biological layers, was defined by the Biological–Histological Discordance Score (BHDS). Additionally, these investigator-defined composite measures were compared with each of the component variables, with the full set of histopathological features, and with increasingly broad clinicobiological models. A variety of established statistical and machine learning methodologies were employed as supplementary analytical tools to assess whether similar organizational principles governing nodal risk could also be identified through prediction, explanation, stability, uncertainty, similarity, network, and unsupervised analyses. Therefore, while this study did not propose a new classification methodology, its methodological contribution arose primarily from the biologically structured nature of its dimensionality reduction and comparative assessments.

Regional LNM confirmed through histopathology was chosen as the primary endpoint because it was more reliably documented than final melanoma stage, which was either not recorded or incompletely recorded and was, therefore, retained for descriptive analyses only. All scores, probabilities, phenotypes, thresholds, and AI-derived outcomes were viewed as internally evaluated exploratory constructs and were not designed to replace sentinel lymph-node biopsy, histopathological staging, molecular testing, multidisciplinary evaluation, or clinical judgment. Consequently, when developing and interpreting models for this study, consideration was given to biological plausibility, parsimony, prevention of information leakage, calibration of predictions, assessment of explanation stability and uncertainty, and evaluation of model performance on held-out observations during internal validation.

### 2.1. Study Population and Analytical Domains

Variables retained after removal of direct identifier data and non-analytic data fields were organized into nine separate analytical domains based on demographic characteristics; the anatomical location of the tumor; the surgical treatment (e.g., the type of anesthesia and/or reconstructive procedure); the histopathological characteristics of the tumor; the characteristics of lymph nodes; the presence of comorbidities; the characteristics of systemic biological responses; the clinical course; and the final stage of cancer (Table 1).

The demographic variables consisted of age; sex; and urban versus rural background. Anatomical variables provided information about whether facial, scalp, neck/cervical, trunk, or limb regions were involved. Operative variables provided information about types of anesthesia used and types of reconstructive procedures (primary closure vs. local flaps vs. locoregional flaps vs. skin grafts). The histopathological variables consisted of Breslow thickness; Clark level; ulceration; mitotic activity; lymphovascular invasion (LVI); vertical growth phase (VGP); specific melanoma subtypes; and presence of satellite lesions/in-transit metastasis. Specific melanoma subtypes were categorized into superficial spreading melanoma; nodular melanoma; acral lentiginous melanoma; and in situ melanoma. For the purposes of creating the Histopathologic Aggressiveness Index (HAI), additional coding for nodular melanoma was created to represent this subtype as a binary variable.

The nodal domain included lymphoscintigraphy; identification of the specific nodal basin(s); palpable adenopathy; and histopathological confirmation of LNM. Only those cases with histopathological confirmation of LNM were designated as having LNM; lymphoscintigraphy and other nodal basin-related variables were coded as anatomically mapped locations but not as surrogates for the outcome. Systemic biological variables included serum ionized calcium; serum albumin; total serum calcium; corrected serum calcium; C-reactive protein (CRP); interleukin-6 (IL-6); 25-hydroxyvitamin D; alkaline phosphatase (ALP); fasting glucose; and lactate dehydrogenase (LDH). The duration of hospitalization was classified under clinical course variables. The final disease stage was retained solely for description.

Pathology reports for regional lymph nodes were available for 72 of 73 patients. Twenty-two patients had histopathologically confirmed LNM, and 50 were node-negative; one case lacked documentation regarding nodal status. Thus, all subsequent analyses involving regional LNM were conducted using 72 patients with 22 events. The relatively small size of this cohort was due entirely to the availability of the original dataset in the form of a retrospectively curated registry. No a priori sample size calculation was performed; rather, consideration of the relatively low number of events influenced the selection of simpler, less bias-prone, penalized, and resampling-based methodologies.

In addition to being processed differently relative to one another within different portions of the analytical structure, the 39 variables retained in this study were not entered into each model simultaneously. Instead, four interrelated questions were asked: Did individual traditional variables discriminate nodal status? Could the histopathological signal be reduced to a single interpretable measure of aggressiveness? Was there additional informative value in combining systemic inflammatory/metabolic information or measuring discordance between histology and biology beyond what was captured by that single aggressiveness index? And could complementary supervised and unsupervised analyses recover a similar clinicobiological organization?

Genomic and transcriptomic data were unavailable; accordingly, neither BRAF mutations nor any other forms of genomic sequencing information were integrated into this analysis. The framework is, therefore, best characterized as clinicopathological/systemic biological rather than genomically validated.

### 2.2. Data Curation, Transformation, and Missing-Data Handling

All records had personally identifiable information (PII) removed prior to analysis. Variable names, category labels for categorical variables, usage of commas or periods as decimal separators for numeric variables, binary encoding schemes for binary variables, and representations of missing data were normalized. Missing-data representations were transformed into a consistent missing-data format rather than assigned arbitrary numerical values. Variables that could be logically interpreted as binary variables (ulceration; LVI; VGP; satellite lesions/in-transit metastases; nodular subtype; palpable adenopathy; etc.) were encoded as 0/1 values. Clark level was treated as an ordinal variable.

Laboratory values measured as continuous variables underwent review for consistency in units of measurement; biological plausibility; degree of distributional skewness; outlier values exceeding normal laboratory ranges; reporting limits below measurable values; and patterns related to missingness. Laboratory values that fell below reporting limits were replaced according to predefined reporting-limit rules for sensitivity evaluations. Albumin-corrected calcium was computed from total serum calcium using the following equation: Corrected calcium (mg/dL) = Total calcium (mg/dL) + 0.8 × [4.0 − Serum albumin (g/dL)]. This formula adjusts total calcium for variations in serum albumin concentration. Continuous right-skewed variables, including C-reactive protein, interleukin-6, Breslow thickness, mitotic activity, and lactate dehydrogenase, were evaluated after logarithmic transformation using log(1 + measured value).

When standardized inputs were needed for computation (e.g., calculating z-scores), mean and standard deviation statistics were computed on training data alone and subsequently applied to the held-out observations.

All eight HAI components were completely available, thereby enabling direct computation of HAI for 73/73 subjects. Missingness was limited for CRP (n = 2), IL-6 (n = 2), and LDH (n = 1), but was greater for vitamin D (n = 14); glucose, ALP, and corrected calcium were present in all subjects. Thus, IMDI/BHDS could be computed without the need for imputation in 59/73 subjects (80.8%). For models requiring incomplete systemic measurements to be imputed for internal validation purposes, continuous variables were imputed using medians and ordinal/binary variables using modes, with both computed only within the corresponding training folds. The resulting fitted imputation rule was then applied to the held-out subject. Complete-case sensitivity analyses were performed when possible.

Neither nodal status nor final disease stage was imputed.

Any operation dependent upon data that might provide information crossing over from training data into held-out data—including imputation; transformation; standardization; estimation of coefficients for derived scores; normalization of scores; and model fitting—was strictly prohibited from accessing held-out data during cross-validation.

### 2.3. Biologically Structured Feature Engineering

As mentioned earlier, HAI, IMDI, and BHDS were specifically designed for use with this study’s design and structure and, thus, had not been previously validated as melanoma-staging tools. Thus, we define each metric fully so that the reader may replicate the findings presented.

HAI measures local tumor aggressiveness across eight biological domains: Breslow thickness, mitotic activity, Clark level, ulceration, lymphovascular invasion (LVI), vertical growth phase (VGP), nodular subtype, and satellitosis/in-transit metastasis. To create a linear predictor for predictive analysis of HAI, we created a formula based on the full-cohort linear predictor: logit(p) = −4.12 + 1.84 · log(Breslow + 1) + 1.21 · log(mitoses + 1) + 0.67 · Clark + 1.53 · ulceration + 1.92 · LVI + 0.21 · VGP + 1.09 · nodular subtype + 0.43 · satellitosis. Binary variables were coded 0/1 to represent the presence/absence of each factor, and Clark level was coded as ordinal. We note that these coefficients represent the best-fit full-cohort model and are provided for replication purposes. In LOOCV, coefficients were estimated again in each training fold rather than being imposed on the held-out patient.

To transform the linear predictor into a 0–100 scale as needed for comparison (with the “i” subscript referring to the patient under consideration and η representing the patient’s linear predictor), we used the following equation: HAI_i = 100 × (η_i − η_min,train)/(η_max,train − η_min,train). Higher HAI values, thus, represent greater histopathological aggressiveness.

IMDI reflects systemic inflammatory–metabolic dysregulation through seven systemic biomarkers: CRP, IL-6, LDH, fasting blood glucose, ALP, corrected serum calcium, and vitamin D. The representative full-cohort linear predictor for IMDI was as follows: logit(p) = −1.89 + 0.24 · log(CRP + 1) + 0.31 · log(IL-6 + 1) + 0.18 · log(LDH + 1) + 0.09 · glucose + 0.06 · ALP + 0.12 · corrected calcium − 0.27 · vitamin D. Since the coefficient for vitamin D was negative, no reciprocal transformation was applied. Like HAI, all operations for estimating coefficients for HAI and IMDI—i.e., imputing missing values as necessary, transforming skewed values, standardizing predictors as necessary, and normalizing scores—were repeated in each of the 72 LOOCV training folds before applying those results to predict the held-out patient. Therefore, higher values of IMDI indicate higher degrees of model-defined systemic dysregulation.

BHDS calculates the amount of discrepancy between the local and systemic axes as BHDS_i = |rank(HAI_i) − rank(IMDI_i)|/(n − 1). Because BHDS is nondirectional, it ranges from 0 to 1. When BHDS = 0, the ranks for HAI_i and IMDI_i are identical across both axes. Therefore, as BHDS approaches 1, there is increasing disagreement regarding the relative rank position between the local and systemic axes.

Although BHDS indicates the magnitude of disagreement between the local and systemic axes, it does not specify its directionality. We, therefore, developed an exploratory phenotype classification for illustrative purposes: Low-Risk Concordant—HAI ≤ 53.6 and IMDI ≤ 34.6; Histology-Dominant—HAI > 53.6 and IMDI ≤ 34.6; Biology-Dominant—HAI ≤ 53.6 and IMDI > 34.6; and High-Risk Concordant—HAI > 53.6 and IMDI > 34.6. This phenotypic classification was used solely to illustrate the direction of clinicobiological discordance and was not used to construct any of our primary models.

Additional model-derived quantities included the Nodal Metastatic Probability Score (NMPS), the Melanoma Clinicobiological Risk Score (MCRS), and the Explanation Stability Index (ESI), as summarized in Table 2.

### 2.4. Internal Validation and Comparative Modeling

Internal validation was critical due to the large number of candidate variables relative to sample size. However, given the relatively small number of evaluable patients (n = 72), a traditional random 80/20 training/validation split could not be utilized, as such a split would result in too few observations/events in the validation cohort to reliably estimate model performance. Therefore, LOOCV was used primarily for internal validation.

At each iteration of the LOOCV process (i.e., n = 72), one patient was withheld from model development, and all subsequent model development steps (e.g., imputation of missing values; transformation of skewed variables; standardization of predictors; estimation of coefficients for HAI and IMDI within each training fold; normalization of scores; and fitting of each model) were conducted utilizing only the training-fold data prior to generating an out-of-fold prediction for the withheld patient. Through repetition of this process across all patients, 72 out-of-fold predictions were generated for each model and used to derive estimates of model performance.

Bootstrap resampling with replacement (n = 1000 repetitions) was used to quantify variability in several key estimates and to assess stability in feature selection and threshold placement. Permutation testing (n = 1000 permutations of nodal status) was used to test hypotheses concerning cross-validated discrimination via generation of a null distribution against which we compared the observed cross-validated discrimination.

Bias-reduced/Firth logistic regression was utilized in conjunction with penalized logistic regression as the primary modeling strategy for deriving associations in the setting of limited event counts and possible partial separation.

In addition to logistic regression models, secondary nonlinear approaches were considered, including Random Forest, Extra Trees, Gradient Boosting Machine, AdaBoost Classification Tree, Shallow Classification Tree, Radial Basis Function Support Vector Machine, K-Nearest Neighbors Classifier, and Gaussian Naïve Bayes.

Model complexity was constrained; e.g., Random Forest models had a maximum depth of 3 and a minimum node size of 5.

Unrestricted hyperparameter optimization and broad AutoML searches were excluded.

We determined how much information was retained via biological aggregation by evaluating HAI in relation to each component individually, as well as to a conventional model that incorporated all eight histopathological variables separately. Likewise, we assessed the incremental value added by considering systemic factors by comparing HAI alone versus HAI + IMDI + BHDS, as well as larger models including demographics, anatomical location, comorbidities, and palpable adenopathy.

Additionally, we evaluated whether systemic inflammatory–metabolic dysregulation modulated the relationship between local aggressiveness (as reflected by HAI) and LNM by examining an exploratory HAI × IMDI interaction term.

Finally, a simple classification tree was independently developed to generate a clinically understandable partition of HAI. The classification tree was developed across the entirety of the analytical cohort and then simplified into a rule-based classifier. While this partition was developed from the same full cohort rather than being identified within cross-validation, it was treated as an exploratory cohort-derived rule rather than an internally validated clinical cutoff. The internal stability of this partition was evaluated across 1000 bootstrap resamples by examining the distributions and frequencies of recovered root-split values.

### 2.5. Model Evaluation and Incremental Predictive Value

Evaluation metrics were not used as objective functions during model building. Each model was built to optimize its respective likelihood function or algorithm-specific loss/objective function; subsequent evaluation was based upon out-of-fold predictions.

For each model, we evaluated discrimination using the receiver operating characteristic area under the curve (ROC AUC) and precision–recall area under the curve (PR-AUC). The latter was incorporated since the primary outcome consisted of only 22 positive and 50 negative cases. Confidence intervals (CIs) were computed for all key estimates; furthermore, correlated ROC AUCs were compared using DeLong tests where applicable. Differences in the ROC AUC, PR-AUC, and Brier score were also reported between paired models with CIs.

The Brier score was employed as an evaluation metric for assessment of probabilistic prediction error; calibration was assessed using calibration intercepts and slopes, the Integrated Calibration Index (ICI), and graphical representations depicting calibration. Discrimination and calibration were evaluated jointly.

Performance characteristics relevant to thresholds were assessed using sensitivity, specificity, positive predictive value (PPV), negative predictive value (NPV), balanced accuracy, the F1 score, and the Matthews correlation coefficient (MCC). Wilson CIs were computed for sensitivity, specificity, PPV, and NPV related to exploratory HAI rules developed from the same cohort, providing the corresponding threshold; thus, they were treated descriptively rather than being viewed as prospective performance assessments.

The additional predictive value contributed by each model beyond what was accounted for by HAI was analyzed through paired comparisons among models combined with continuous net reclassification improvement (NRI) and integrated discrimination improvement (IDI). NRI evaluates changes in relative rankings of patient risks, while IDI evaluates changes in mean predicted probability separation between endpoint groups. Both analyses were assessed based on CIs and *p*-values instead of merely analyzing the signs of their point estimates.

Decision curve analysis compared an HAI-centered model, broader integrated models, and Random Forest with treat-all and treat-none strategies at various exploratory threshold probabilities, including 10%, 20%, 30%, 40%, and 50%. Net benefits obtained from decision curve analysis were interpreted as cohort-level decision-theoretic information rather than as evidence warranting implementation in clinical settings.

### 2.6. Explainability, Stability, and Uncertainty

Rather than simply describing explainability in qualitative terms, we sought to evaluate empirical aspects thereof as an attribute of our proposed methodology.

Global contributions of predictor j were determined using SHAP (Shapley Additive Explanations), applied to analyze the principal integrated explanation model, which was an integrated Firth-penalized logistic regression model including HAI, BHDS, age, IMDI, palpable adenopathy, anatomical site, and comorbidity information. SHAP analysis was performed on the final model fitted to all 72 patients with documented nodal status and was used to characterize the behavior of the fitted model separately from the out-of-fold predictions used to estimate predictive performance. Contributions from predictor j to overall model predictions were quantified as mean |SHAP_j| = Σ_i |SHAP_ij|/n; contributions relative to all predictors k were quantified as mean |SHAP_j|/Σ_k mean |SHAP_k| × 100%. The directionality of SHAP values reflected whether predictor values resulted in upward or downward shifts in model output. SHAP distribution summaries for HAI were further quantified according to nodal status. SHAP values reflected model-based attribution rather than direct causality.

Nonlinear feature importance estimates for assessing variable contributions were generated through an independent full-feature Random Forest analysis.

The stability and repeatability of explanations were assessed across 1000 bootstrap resamples through documentation of how frequently major variables ranked first, within the top three, or within the top five, as well as their median ranking and IQR. Finally, overlap between the three most important explanatory variables was summarized using ESI, distinguishing explanatory anchors with high stability across resampled cohorts from those with lower stability.

Separately from explaining predictions, prediction uncertainty was assessed using conformal prediction at a nominal target coverage rate of 90%. Conformal prediction allowed either a singleton prediction set or a two-label set if uncertainty remained substantial. We report overall coverage, coverage stratified by nodal status, prediction-set sizes, and the proportions of singleton and two-label sets.

### 2.7. Phenomapping, Similarity Retrieval, and Cross-Layer Structure

To assess whether the supervised results could be recovered through unsupervised methods (phenomapping) and relational methods (similarity retrieval), we applied both methods to determine whether the organization found through supervised machine learning could also be recovered through different methodologies that did not organize the data around nodal status. To evaluate these unsupervised and relational analyses, we utilized a combination of clinical, histopathological, and systemic biological variables through phenomapping. We also evaluated hierarchical clustering, partitioning around medoids, k-prototypes, and low-dimensional visualization to assist with our exploratory assessments. Once candidate structures for comparison were identified using silhouette analysis, we evaluated the stability of the retained two-cluster structure using bootstrap Jaccard similarity. After evaluating cluster formation through the previously mentioned methods, we then evaluated nodal status along with HAI and IMDI distributions.

The melanoma “digital twin” component was implemented as similarity-based case retrieval rather than as a generative simulator. We represented patients through a variety of clinical, histopathological, and biological characteristics. Additionally, we utilized Gower distance to incorporate continuous, ordinal, binary, and categorical data into a single similarity metric. Through Gower distance, we were able to retrieve the closest-neighbor cases to each index patient and their respective nodal/clinicobiological profiles. This method was viewed as a case analogy and distribution support tool rather than as a standalone classifier.

Through Spearman rank correlation, we assessed cross-layer relationships, particularly HAI–BHDS, HAI–IMDI, and IMDI–BHDS. Additionally, we created a network representation in which variables became nodes and associations of |ρ| > 0.30 between variables became weighted edges. We calculated weighted degree to quantify connectivity across the entire network. Betweenness centrality was used to identify variables located at the intersection of local histopathology and systemic biological domains. Network measures were interpreted as structural associations within the observed cohort rather than as causal biological pathways.

### 2.8. Bias Control, Reproducibility, and Scope of Inference

Throughout the workflow, we incorporated bias-control procedures. We strictly defined histopathologically confirmed lymph-node metastasis (LNM) as the outcome variable. We also excluded final disease stage from our primary predictive model. All data-dependent preprocessing was performed within the corresponding leave-one-out cross-validation (LOOCV) training fold. Model complexity was also restricted in consideration of the 22 endpoint events. Our primary performance metrics were derived from out-of-fold predictions rather than from apparent fit of the model to the full cohort. We employed bootstrap resampling, permutation testing, calibration analysis, decision curve analysis, and explanation stability assessments as complementary approaches to internal validation.

Because the cohort-derived HAI threshold was intentionally derived separately from the internally validated continuous model, only its internal stability was evaluated through bootstrap analysis. Therefore, this threshold was not interpreted as an externally validated clinical cutoff.

Limitations in the scope of inference included, but were not limited to, the retrospective single-center design, limited sample size, 22 node-positive events, incomplete systemic biomarker profiles, and absence of genomic information. Therefore, HAI, IMDI, BHDS, directional clinicobiological phenotypes, NMPS, MCRS, HAI thresholds, similarity retrieval, conformal outputs, and unsupervised phenotypes should be regarded as internally evaluated research constructs. Ultimately, independent multicenter validation, recalibration, and prospective evaluation will be necessary before any component can be considered for clinical decision support.

All analyses were performed locally using Python v3.14.7 (Python Software Foundation, Wilmington, DE, USA) and R v4.6.1 (R Foundation for Statistical Computing, Vienna, Austria), together with established statistical and machine-learning packages for regression, validation, calibration, explainability, clustering, and uncertainty analysis. Computations were performed on a 64-bit workstation with an Intel Core i7-13620H processor (Intel Corporation, Santa Clara, CA, USA) and 16 GB RAM.

## 3. Results

### 3.1. Cohort Characteristics and Primary Endpoint

This study included 73 patients with malignant melanoma, with a median age of 66 years (interquartile range [IQR]: 49–73 years). Documentation regarding sex was available in 68 patients (33 female and 35 male); 53 patients had an urban background, and 20 had a rural background. In terms of anatomical site location, information was available in 67 patients, and the most common recorded locations included the trunk (n = 42) and extremities (n = 23), followed by the scalp (n = 5), face (n = 4) and cervical region (n = 1). These anatomical indicators were recorded as non-mutually exclusive binary variables; therefore, their summed counts exceeded the number of patients with documented site information.

Clark level IV was the most common invasion category (n = 45), followed by Clark levels III (n = 17), V (n = 5), II (n = 3), I (n = 2), and 0 (n = 1). The median Breslow thickness was 3.00 mm (IQR: 1.20–5.70 mm), and the median mitotic activity was 5 mitoses/mm^2^ (IQR: 2.0–13.5). Superficial spreading melanoma was the most frequently occurring melanoma subtype (n = 42), followed by nodular melanoma (n = 27; 37.0%), melanoma in situ (n = 3), and acral lentiginous melanoma (n = 1).

Histopathological nodal status was available for 72/73 patients. Twenty-two were node-positive, and 50 were node-negative. One patient without documented nodal pathology was excluded from primary endpoint analysis. Final disease stage was documented in 24 patients.

The availability of systemic biomarkers varied from 59/73 for vitamin D to complete availability for glucose, alkaline phosphatase (ALP), and corrected calcium. The median values of the available biomarkers were as follows: CRP, 0.40 mg/dL; IL-6, 3.01 pg/mL; vitamin D, 23.98 ng/mL; LDH, 172.5 IU/L; glucose, 98 mg/dL; ALP, 64 U/L; and corrected calcium, 9.06 mg/dL. The median hospital stay was 6 days among the 52 patients with available data for this variable.

### 3.2. Nodal Metastasis Is Associated Predominantly with Local Histopathological Aggressiveness

Node-positive melanomas displayed significantly greater local histopathological aggressiveness than node-negative melanomas. For example, the median Breslow thickness was 5.85 mm in node-positive patients compared with 1.80 mm in node-negative patients (*p* < 0.001), and the median mitotic activity was 14 versus 3 mitoses/mm^2^, respectively (*p* < 0.001). Additionally, ulceration was found in 19/22 node-positive patients compared with 20/50 node-negative patients (odds ratio [OR] = 9.5; *p* < 0.001), lymphovascular invasion (LVI) was seen in 10/22 node-positive patients and 3/50 node-negative patients (OR = 13.1; *p* < 0.001), and nodular melanoma was identified in 15/22 node-positive patients and 12/50 node-negative patients (OR = 6.8; *p* < 0.001). Although palpable adenopathy was less frequent than some of the other features listed above, it was also associated with nodal involvement (5/22 node-positive patients compared with 2/50 node-negative patients; OR = 7.1; *p* = 0.025). While satellitosis/in-transit metastases were seen in 4/22 node-positive patients and 3/50 node-negative patients (OR = 3.5; *p* = 0.190), vertical growth phase (VGP) demonstrated limited separation between the two groups because of its high prevalence in both.

Differences in systemic features were much smaller. For instance, median IL-6 levels were 3.65 pg/mL in node-positive patients and 2.93 pg/mL in node-negative patients (*p* = 0.082), while median vitamin D levels were 19.59 ng/mL and 24.13 ng/mL, respectively (*p* = 0.130). Similarly, median glucose levels were 105.5 mg/dL in node-positive patients and 95.5 mg/dL in node-negative patients (*p* = 0.129). As with the remaining systemic factors, there was little evidence of clear univariate separation.

Median Histopathologic Aggressiveness Index (HAI) values were notably higher in node-positive patients (67.8 vs. 45.3; *p* < 0.001), whereas median Inflammatory–Metabolic Dysregulation Index (IMDI) values were only modestly different (39.1 vs. 33.7; *p* = 0.169), and median Biological–Histological Discordance Score (BHDS) values were similar between groups (0.26 vs. 0.29; *p* = 0.533).

### 3.3. HAI Retains Multivariable Histopathological Information While Exceeding the Strongest Individual Feature

Among the individual HAI components, Breslow thickness provided the greatest discrimination of nodal metastasis, with a ROC AUC of 0.852 (95% confidence interval [CI]: 0.758–0.921), followed by mitotic activity at 0.831 (95% CI: 0.733–0.907), ulceration at 0.791 (0.681–0.876), LVI at 0.769 (0.654–0.862), nodular subtype at 0.741 (0.624–0.838), Clark level at 0.718 (0.598–0.818), VGP at 0.598 (0.478–0.710), and satellitosis/in-transit metastases at 0.583 (0.462–0.697).

HAI demonstrated a ROC AUC of 0.889 (95% CI: 0.814–0.948), which exceeded the discrimination obtained using Breslow thickness alone by ΔAUC = +0.037 (95% CI: +0.008 to +0.066; *p* = 0.014). In comparison, a model incorporating all eight histopathological variables separately achieved a ROC AUC of 0.901 (95% CI: 0.831–0.948), PR-AUC of 0.728 (0.602–0.834), Brier score of 0.132 (0.098–0.170), sensitivity of 95.5% (77.2–99.2%), specificity of 82.0% (68.6–90.6%), and balanced accuracy of 0.888. HAI performance was, therefore, close to that of the complete histopathological model while exceeding that of the strongest individual feature (Table 3).

### 3.4. Direction, Rather than Magnitude, of Histology–Biology Discordance Is Informative

Although BHDS could not distinguish between the two nodal groups based on the magnitude of discordance, there was a statistically significant relationship when considering the direction of HAI and IMDI. That is, when medians were considered for both HAI (53.6) and IMDI (34.6), 18 patients had low-risk concordant histology–biology scores, 31 patients had histology-dominant scores, 14 had biology-dominant scores, and nine patients had high-risk concordant scores. Among these patients, 0/18 (0%), 16/31 (51.6%), 2/14 (14.3%), and 4/9 (44.4%) had lymph-node metastasis (LNM), respectively (Fisher’s exact test *p* < 0.001).

Correlation analysis indicated that HAI and IMDI were moderately correlated (ρ = 0.41; *p* < 0.001), whereas correlations of HAI and IMDI with BHDS were weaker (ρ = 0.23; *p* = 0.052 for HAI vs. BHDS; ρ = 0.31; *p* = 0.009 for IMDI vs. BHDS). Also, the interaction term between HAI and IMDI was not significant (OR per joint 10-unit increase = 0.96; 95% CI: 0.89–1.04; *p* = 0.31). Therefore, although the magnitude of discordance provided little information regarding the endpoint, the direction of discordance provided useful information for identifying clinicobiological states with differing LNM distributions (Table 4).

### 3.5. HAI Remains the Principal Adjusted and Internally Validated Predictor

HAI remained the variable most strongly associated with LNM in a bias-reduced multivariable model adjusting for potential confounders. The odds ratio per 10-point increase in HAI was 2.74 (95% CI: 1.58–4.75; *p* < 0.001). Neither IMDI (OR per 10-unit increase = 1.13; 95% CI: 0.72–1.79; *p* = 0.598) nor BHDS (OR per 0.10-unit increase = 0.95; 95% CI: 0.64–1.39; *p* = 0.774) demonstrated independent associations with LNM. Age also showed no independent association (OR per 10-year increase = 0.82; 95% CI: 0.51–1.32; *p* = 0.409), while the presence of palpable adenopathy demonstrated a positive but imprecise estimate (OR = 2.18; 95% CI: 0.25–19.40; *p* = 0.484).

**Figure 1:**
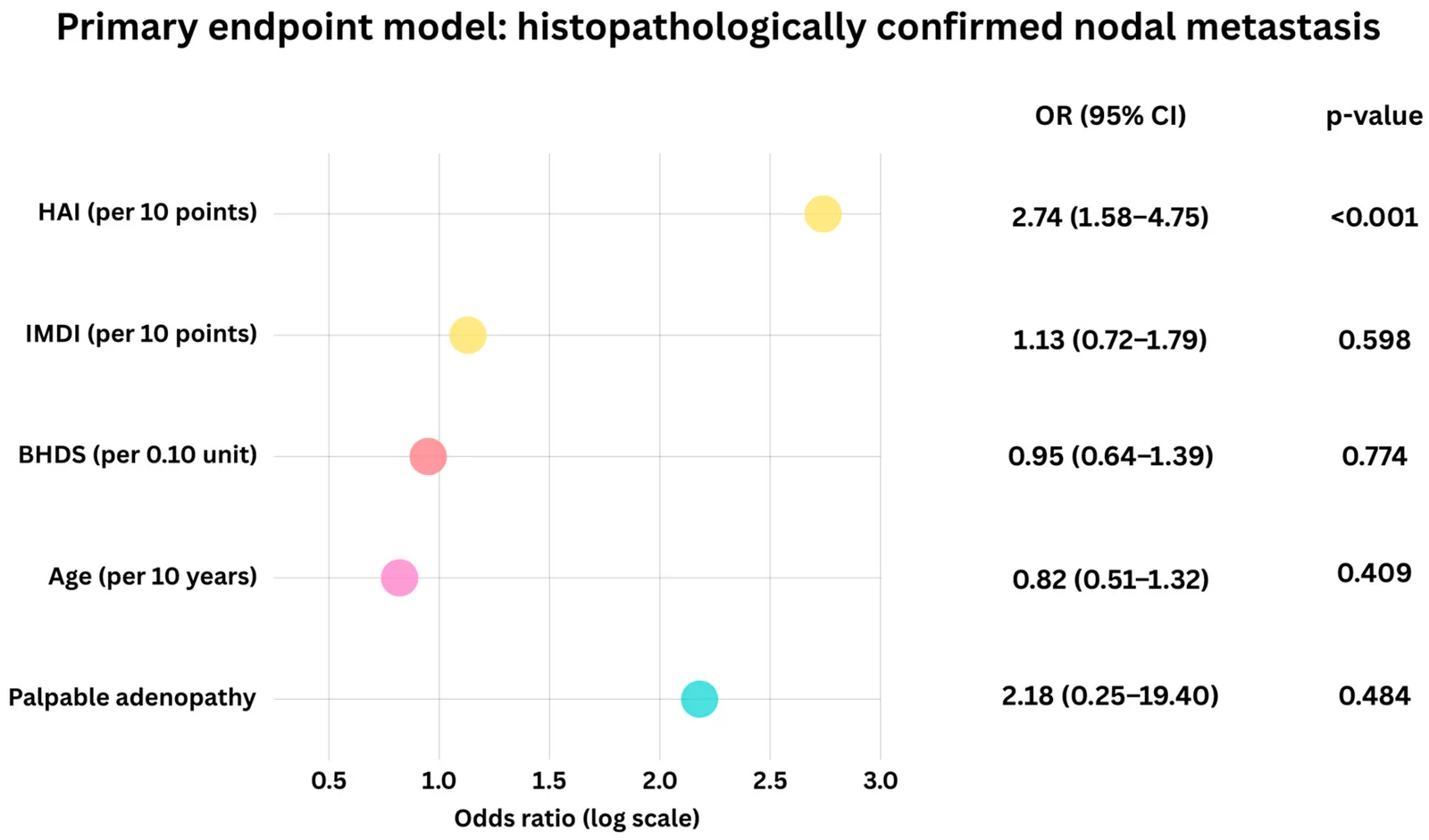
Primary endpoint model for histopathologically confirmed lymph-node metastasis. Effect estimates from the bias-reduced multivariable model. HAI retained the strongest adjusted association with LNM (OR: 2.74 per 10-point increase; 95% CI: 1.58–4.75), whereas confidence intervals for the remaining predictors included the null.

The performance metrics for the apparent full cohort are illustrated in Figure 1, where ROC AUC = 0.899 and Brier score = 0.123.

Similarly, LOOCV results using HAI alone produced comparable performance metrics; thus, ROC AUC = 0.889 (95% CI: 0.814–0.948), PR-AUC = 0.706, Brier score = 0.137, balanced accuracy = 0.845, F1 score = 0.755, and MCC = 0.641); furthermore, at the threshold used to evaluate the cross-validated model, the sensitivity was 90.9%, and the specificity was 78.0%. These threshold-dependent measures are distinct from those reported in Section 3.6 for the full-cohort-derived HAI > 58.6 rule.

Also, the predictive signal generated by HAI exceeded chance expectations. Among 1000 random permutations of the endpoint vector, the mean null ROC AUC was 0.512 (95% CI: 0.462–0.562); the 95th percentile of the null distribution was 0.663. Therefore, the observed-minus-null difference was 0.377 (*p* = 0.002).

In addition to having a strong predictive signal, it should be noted that the addition of IMDI and BHDS to the model did not provide additional predictive benefit: HAI + IMDI + BHDS produced ROC AUC = 0.879, PR-AUC = 0.701, and Brier score = 0.143. Furthermore, the added predictors resulted in ΔAUC = −0.010 (95% CI: −0.031 to +0.011; *p* = 0.34), ΔPR-AUC = −0.005 (95% CI: −0.029 to +0.019), and ΔBrier = +0.006 (95% CI: −0.004 to +0.016). Finally, continuous NRI was +0.331 (95% CI: −0.042 to +0.704; *p* = 0.081), whereas IDI was −0.039 (95% CI: −0.087 to +0.009; *p* = 0.112). Thus, there was no compelling evidence that the addition of IMDI and/or BHDS substantially improved average risk separation beyond what could be achieved through HAI alone. For further reference, see Table 5.

Overall performance was strongest when centered around HAI or when Random Forest was used to generate predictions. Increasing model complexity did not produce consistent advantages over HAI with respect to discrimination, prediction error, classification performance, or parsimony.

These findings were also supported by DCA. At threshold probabilities of 10%, 20%, 30%, 40%, and 50%, HAI had net benefits of 0.312, 0.226, 0.148, 0.079, and 0.021, respectively; the integrated model demonstrated net benefits of 0.287, 0.201, 0.122, 0.053, and −0.008; and Random Forest demonstrated net benefits of 0.289, 0.201, 0.119, 0.048, and −0.012. Treat-all exhibited net benefits of 0.218, 0.132, 0.064, 0.011, and −0.032, while treat-none produced a net benefit of 0 at every threshold probability examined.

Lastly, HAI provided greater net benefit than treat-all across threshold probabilities of approximately 0.07–0.44.

### 3.6. The HAI > 58.6 Partition Is Readable and Internally Stable but Cohort-Specific

At an HAI cutoff of >58.6, 33 patients had values > 58.6, including all 22 node-positive patients and 11 node-negative patients, whereas 39 patients had values ≤ 58.6, all of whom were node-negative. Therefore, sensitivity was 100% (95% CI: 84.6–100.0%), specificity was 78.0% (64.8–87.2%), PPV was 66.7% (49.6–80.2%), NPV was 100% (92.3–100.0%), balanced accuracy was 0.890 (0.823–0.936), F1 score was 0.800, and MCC was 0.721.

Additionally, the partition was internally stable, as evidenced by bootstrap analysis using 1000 resamples, with a median recovered split of 58.4 (IQR: 56.2–60.1) and a 2.5th–97.5th percentile interval of 52.3–64.8. Eighty-four percent of these resamples produced splits between 55 and 62.

**Figure 2:**
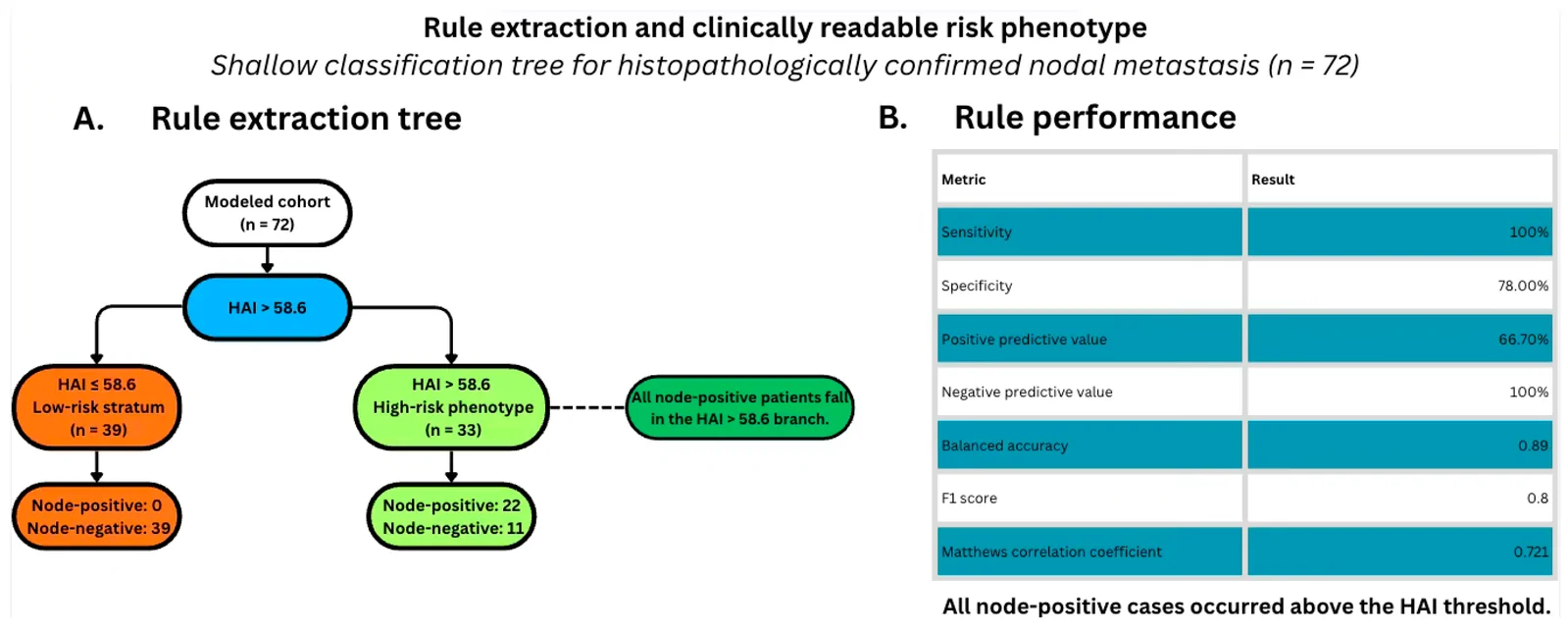
HAI-based exploratory partition of nodal risk. The first root split occurred at HAI > 58.6. All 22 node-positive patients were above this value, while all 39 patients at or below it were node-negative. Bootstrap resampling yielded a median split of 58.4 (IQR: 56.2–60.1), supporting internal stability of the partition location without constituting external validation.

Thus, while none of the node-positive patients in this dataset exhibited HAI values ≤ 58.6, the 58.6 threshold represents a cohort-derived exploratory partition rather than a clinically established cutoff, as illustrated in Figure 2.

### 3.7. SHAP and Bootstrap Resampling Identify HAI as the Most Stable Explanatory Feature

Hence, we employed SHAP to provide each predictor with a contribution value for each observation in the dataset. HAI accounted for the highest mean absolute SHAP value (0.184), equivalent to 35.8% of the total normalized attribution. HAI was followed by BHDS (0.092 or 17.9%), age (0.071 or 13.8%), IMDI (0.063 or 12.3%), palpable adenopathy (0.048 or 9.4%), anatomical site (0.035 or 6.8%), and comorbidity (0.021 or 4.0%). Higher HAI values were associated with increased predicted probability of nodal involvement, consistent with the direction observed for Breslow thickness, mitotic activity, ulceration, lymphovascular invasion, and nodular subtype. IMDI showed a weak positive association; BHDS displayed a context-dependent pattern; age exhibited a mild negative relationship; and palpable adenopathy showed a positive relationship. Patients with positive nodes displayed a median HAI SHAP value of +0.21 (interquartile range [IQR]: +0.14 to +0.29), while those with negative nodes displayed a median HAI SHAP value of −0.09 (IQR: −0.19 to +0.02).

Consistent with the results above, resampled estimates were highly stable. Across all 1000 bootstrap samples, HAI maintained its rank as the most important variable and was, therefore, also located within the top three variables in 100% of samples. The median rank of HAI was 1.0 (IQR: 1.0–1.0). IMDI occupied the top three ranks in 74% of bootstrap samples and had a median rank of 3.0 (IQR: 2.0–4.0). Age occupied the top three ranks in 60% of bootstrap samples and had a median rank of 4.0 (IQR: 3.0–5.0). BHDS occupied the top three ranks in 52% of bootstrap samples, with a median rank of 3.5 (IQR: 2.0–5.0). Palpable adenopathy was located within the top five variables in 78% of bootstrap samples and had a median rank of 5.0 (IQR: 4.0–6.0). The top-three ESI was 0.604.

There was a consistent ordering of variables between Random Forest feature importance and SHAP estimates. The full-variable Random Forest analysis resulted in a hierarchical ordering of the variables: HAI, 0.284; BHDS, 0.162; age, 0.118; IMDI, 0.104; palpable adenopathy, 0.081; anatomical site, 0.062; comorbidity, 0.041; and all remaining features combined, 0.148. The importance of HAI was approximately 1.75-fold greater than that of BHDS, the second-ranked individual variable (Table 6).

### 3.8. Cross-Layer and Unsupervised Analyses Preserve Biological Context Without Supplanting HAI

In addition to identifying relationships between histopathological and biological variables with |ρ| > 0.30, we performed a network analysis to explore relationships between local-domain and systemic-domain information. We observed several cross-layer relationships that were consistent with our preceding analyses, indicating that local-domain and systemic-domain information were partially coupled but contributed differently to prediction. Additionally, HAI possessed the greatest weighted degree in the network, while IMDI possessed the greatest betweenness centrality.

Case similarity signals were identified through similarity-based retrieval, with top-one nodal concordance equal to 66.7% and top-three majority-vote accuracy equal to 73.6%. Eight patients fell outside the central mass of patients characterized by their similarity to the registry profile (>90th percentile of Gower distance), including two patients with lymph-node metastasis, suggesting that similar cases can be retrieved through Gower-distance-based similarity measures; however, this method was less discriminative than the primary supervised models.

At a nominal conformal coverage of 90%, empirical coverage was 93.1%. On average, prediction sets contained 1.47 labels per patient (range: 1–2). Singleton label sets were assigned to 38 patients, while two-label sets were assigned to 34 patients.

Singleton label sets were assigned to 90.9% (20/22) of patients with lymph-node metastasis and two-label sets to 9.1% (2/22); among patients without lymph-node metastasis, singleton label sets were assigned to 36.0% (18/50), and two-label sets to 64.0% (32/50).

Conformal coverage was achieved in 95.5% (21/22) of patients with lymph-node metastasis and in 92.0% (46/50) of patients without lymph-node metastasis.

Using unsupervised learning techniques, we identified patient phenotypes that differed in terms of lymph-node metastasis prevalence as well as HAI and IMDI values. The two-group solution showed modest separation (silhouette score: 0.197) but relatively high bootstrap stability (Jaccard index: 0.82). Phenotype 1 consisted of 30 patients, among whom 19 (63.3%; 95% CI: 45.5–78.1%) had lymph-node metastasis, while Phenotype 2 consisted of 42 patients, among whom 3 (7.1%; 95% CI: 2.5–19.0%) had lymph-node metastasis.

We found that median HAI values were significantly higher in Phenotype 1 than in Phenotype 2 [68.2 (IQR: 58.4–76.1) vs. 43.1 (IQR: 34.2–52.8), respectively; *p* < 0.001], and similarly for IMDI [37.8 (IQR: 30.2–45.6) vs. 31.7 (IQR: 24.8–38.9), respectively; *p* = 0.039]. Thus, since lymph-node metastasis did not define either group, enrichment of lymph-node metastasis in Phenotype 1 represents an exploratory outcome-independent observation consistent with the supervised analyses described previously.

### 3.9. Integrated Result

Multiple analyses revealed common patterns regarding the relationship between the measured variables and nodal involvement. Notably, HAI emerged as the strongest discriminator among the principal constructed variables, remained the strongest predictor after adjustment for other factors, accounted for the largest SHAP attribution, and consistently ranked first across bootstrap iterations. Unsupervised and network analyses further identified a clinicobiological organization consistent with greater histopathological aggressiveness being associated with a higher prevalence of lymph-node involvement.

Meanwhile, systemic inflammatory–metabolic variables represented a different type of information from HAI. Neither IMDI nor BHDS provided additional predictive benefit beyond HAI alone; however, systemic inflammatory–metabolic information continued to demonstrate relationships with histopathological tumor state, formed directional phenotypes with varying levels of lymph-node metastasis, connected histopathological states to systemically derived states in cross-layer networks, and contributed modestly to the higher-risk phenotype identified through unsupervised analysis.

Thus, this collection of analyses allows us to distinguish predictive importance from contextual relevance: within this cohort, nodal metastatic propensity was predominantly organized along a dimension of measured histopathological aggressiveness, whereas systemic inflammatory–metabolic information primarily represented inter-patient clinicobiological heterogeneity rather than providing measurable improvement in discrimination of nodal metastatic status.

## 4. Discussion

### 4.1. Principal Findings and Conceptual Contribution

The primary contributions of this study include the development of HAI (Histopathologic Aggressiveness Index) to compress multiple melanoma-related variables into a biologically coherent construct and the demonstration that such constructs can retain information carried by their individual components [8]. The results indicate that HAI performs at least as well as each of its individual constituents and approaches the performance of the complete histopathological model, suggesting that a multidimensional pathological phenotype can be represented parsimoniously. Furthermore, these findings were consistently observed using various analytical methodologies, including adjusted modeling, nonlinear benchmarking, explainability, resampling, network analysis, and unsupervised phenotyping [9].

Thus, the convergence of these analyses represents a key aspect of interpreting the framework developed in this study. It is essential to recognize that HAI does not serve as a replacement for existing clinicopathological variables such as Breslow thickness, ulceration, mitotic activity, lymphovascular invasion (LVI), melanoma subtype, or others. Each of these features represents distinct biological processes—including invasion depth, proliferation, tissue destruction, and vascular/lymphatic accessibility—that contribute to melanoma’s ability to disseminate to regional lymph nodes. Instead, the objective of this investigation was to determine whether the combined configuration of these pathological features could be compressed into a single interpretable axis representing local aggressive behavior.

### 4.2. Histopathological Aggressiveness as a Compressed Biological State

It is unlikely that melanoma’s progression toward regional lymph nodes occurs solely because of one specific pathological measurement. For example, a tumor must achieve sufficient invasive depth, proliferative activity, vertical expansion, tissue-destructive capability, and vascular/lymphatic access to allow regional dissemination. Although many of these features are related, they are not interchangeable [10].

The behavior of HAI supports this biological conceptualization. Improved performance over the strongest single pathological feature indicates that HAI captures information present across multiple dimensions. Additionally, the proximity of HAI’s performance to that of the entire histopathological model suggests that much of the relevant information can be captured in a lower-dimensional space. Therefore, we view HAI as a potentially valuable form of biologically structured dimensionality reduction, especially in clinical datasets containing large numbers of correlated pathological variables relative to the number of outcome events.

The conclusions reached regarding HAI should remain conservative. The current data provide no basis for asserting equivalence to comprehensive histopathological assessment. Moreover, there is currently insufficient justification for substituting conventional pathology with derived composite scores. However, the data suggest that the multidimensional pathological state associated with nodal dissemination may be represented through a compact and reproducible axis. Whether such representations remain valid across institutions and populations needs to be investigated through external validation studies [11,12].

### 4.3. Systemic Biology: Context Rather than Incremental Discrimination

Systemic inflammatory–metabolic data were included in this study because melanoma biology encompasses more than the morphological features of tumors. Blood-based inflammatory mediators, lactate dehydrogenase (LDH), glucose metabolism, vitamin D levels, and calcium-related physiological pathways can reflect tumor burden, host response to cancer, immune activation, metabolic stress, nutritional status, and/or comorbidity [13,14].

However, in the present cohort, the systemic component did not contribute additional predictive value after accounting for histopathologically defined aggressiveness. This result does not imply that systemic biology is unimportant. Single-time-point blood-based biomarkers are typically several biological steps removed from the local events necessary for invasion into regional lymph nodes and are subject to variability resulting from infection, medications, nutritional status, perioperative stress, and chronic inflammatory or other disease conditions. Thus, predictive value for contemporaneous regional nodal involvement may be lower than that related to recurrence, systemic progression, or treatment response [15].

Further indication that the systemic and histopathological domains are interdependent comes from the observed correlations between them in both integrated analyses and cross-layer networks, as well as from their contributions to unsupervised patient clustering. While histopathology was primarily responsible for carrying predictive information for the endpoint studied herein, we believe systemic biology characterizes part of a broader tumor–host relationship.

These interpretations are further supported by analyses examining discrepancies between the two types of measurements. While BHDS alone exhibited limited predictive capability for nodal involvement, preservation of the direction of disagreement between histology and biology enabled separation of patients according to nodal distributions. We believe this observation may provide a feasible pathway for investigating patients whose systemic condition appears disproportionate to their local pathological burden [16].

### 4.4. Parsimony, Explainability, and Stability

Studies utilizing clinical AI can become increasingly difficult to interpret when model complexity exceeds the amount of information contained within the underlying dataset. In small oncology cohorts, inclusion of additional predictor variables and incorporation of flexible nonlinear learning mechanisms can enhance apparent fit without creating more stable or transferable representations of risk. We, thus, view the lack of a persistent advantage from increased model complexity as informative rather than disappointing [17,18].

The HAI-centered model produced a favorable balance between discrimination, calibration, and interpretability, while the addition of broader clinicobiological data did not produce a statistically clear improvement in out-of-sample predictive performance. This suggests that the major predictive signal was concentrated along the histopathological axis rather than requiring recovery through high-dimensional nonlinear architectures.

The explanations generated through explainability analyses provide an important layer of understanding with respect to this interpretation. HAI was not simply the top-ranked feature within a given fitted model; it was the dominant explanatory feature across numerous iterations of resampling and was independently prioritized by an additional nonlinear model. We believe this distinction is important because feature importance is more informative when it remains robust to sampling variability [19].

Therefore, our intention was not simply to develop an explainable model; rather, we attempted to investigate whether the explanation itself was reproducible. The agreement among SHAP attribution, bootstrap feature rankings, and Random Forest feature importance indicates that the primacy of HAI was not a product of a single explanation methodology. Secondary variables exhibited less stability in rank and should, thus, be interpreted with greater caution.

### 4.5. Readability, Uncertainty, and Translational Meaning

While the cohort-derived HAI partition demonstrates how a continuous histopathological state may be translated into a clinically readable phenotype, it should not be interpreted as either a treatment threshold or a staging criterion. The value of this partition lies in illustrating that nodal events were concentrated within the higher portion of the histopathological aggressiveness continuum. Since the threshold was derived from the same cohort in which it was evaluated, its apparent classification metrics may be optimistic and require independent validation [20].

Similarly, positive internal net benefit observed through decision curve analysis and the empirical coverage observed through conformal prediction are encouraging but do not demonstrate improved clinical outcomes through use of HAI. While conformal prediction allows uncertainty to remain visible rather than mandating a single response when ambiguity exists in the available evidence, it does not by itself provide a basis for deploying the underlying model into clinical practice.

Lastly, similarity retrieval may offer another avenue for increasing transparency. Comparisons with previously observed clinically similar cases may aid in providing context for clinicians evaluating new patients who fall outside the predominant distribution represented within the training dataset. We, thus, view this as an interpretive support mechanism rather than as an alternative predictive tool to established clinical evaluation [21].

Ultimately, we view the framework presented here as an exploratory decision-support paradigm. We do not intend for HAI, IMDI (Inflammatory–Metabolic Dysregulation Index), BHDS (Biological–Histological Discordance Score), similarity retrieval, or conformal prediction to supplant sentinel lymph-node biopsy (SLNB), pathological staging, molecular diagnostics, multidisciplinary evaluation, or physician clinical judgment.

### 4.6. Convergent Biological Organization Across Supervised and Unsupervised Analyses

An important aspect of the findings from both the supervised and unsupervised analyses is that the structural pattern based on histopathology identified by the supervised models was not exclusive to supervised prediction. Unsupervised phenomapping identified a subgroup of patients who displayed significantly increased histopathological aggressiveness and enrichment for nodal disease; however, because nodal status was not used to group patients into clusters, grouping was based only on the clinicobiological features included in the analysis. While only a modest degree of geometric separation existed between the two groups, and this finding should be considered preliminary given the small sample size, the stability of patients’ assignment to a particular cluster indicates that the observed pattern was not dependent upon only one way of partitioning the data [22].

While both types of methods offered additional views of the data, network analysis showed that HAI (Histopathologic Aggressiveness Index) had the greatest weighted degree of connectivity between nodes, whereas IMDI (Inflammatory–Metabolic Dysregulation Index) had the greatest betweenness centrality, indicating a greater “bridging” position between local and systemic levels of disease. As discussed earlier, neither result provides evidence of a causal relationship; however, they suggest a hierarchical organization in which localized histopathological aggressiveness is central to the structure of local disease, while systemic dysregulation links local processes with a broader host-biological environment [23].

These analyses are informative because multiple analytical techniques independently identified a common hierarchical structure. However, none provides definitive proof that this structure has biological relevance. Each of the analytical techniques used in this investigation is distinct and represents a separate methodological class. Thus, while penalized regression, nonlinear modeling, SHAP attribution, bootstrap stability assessment, phenotyping, and network analysis utilize analytically different methodologies, collectively, they reinforce the biological interpretation of the hierarchy described. Ultimately, however, this interpretation will require independent validation using data from sources outside the cohort studied here [24].

### 4.7. Limitations

Several important limitations exist regarding this study. First, it is retrospective in nature and was conducted at a single institution. Second, only 72 patients could be evaluated for the primary outcome, and only 22 of these patients had positive lymph nodes. Consequently, the uncertainty associated with estimating relationships among multiple variables limits our ability to model complex relationships reliably and increases the probability that some estimated effects are specific to this cohort.

Internal validation strategies, including cross-validation, bootstrap resampling, permutation testing, calibration assessment, and explanation stability assessment, may reduce some forms of optimism. However, they cannot substitute for independent external validation.

HAI was developed using the same population in which it was evaluated. Although HAI was developed separately within each cross-validation training fold, its weighting and transportability must be replicated to evaluate its broader applicability.

Because the threshold defining higher-risk disease in this cohort was determined internally and is likely influenced by optimism, it should serve as a hypothesis-generating starting point for future studies requiring external validation.

In comparison with the various histopathological parameters examined in this study, several systemic parameters were incompletely measured and/or assessed. Specifically, vitamin D was less completely available as a systemic parameter. Systemic measurements were acquired as part of routine clinical care rather than through a prospectively designed protocol for immunometabolic sampling. Hence, the temporal relationship between systemic measurements and surgery, acute inflammation, treatment, nutritional status, and other physiological influences may have affected the quality of the biological signals detected.

Also, final stage, recurrence, progression-free survival, treatment response, and longitudinal changes in biomarkers were insufficiently sampled and, therefore, inadequately represented for meaningful modeling. Because systemic biological layers may be more informative for longitudinal or treatment-related outcomes than for contemporaneous nodal involvement, this limited sampling constrains our ability to determine their relevance over time.

Lastly, no genomic, transcriptomic, or tumor-immune-related variables were available in the dataset. Therefore, we cannot determine whether the dominance of the histopathological axis persists after incorporating BRAF, NRAS, KIT, TERT-promoter, CDKN2A, PD-L1, immune markers, copy-number alterations, or transcriptional signatures. Likewise, while SHAP values, network centrality, clustering, and similarity structures provide informative representations of patterns within the data, they should not be interpreted as direct causal biological relationships. Adding these molecular layers would greatly enhance future investigation of these questions [25].

### 4.8. Future Directions

Clearly, independent multicenter validation should be the first priority for future research before attempting to increase analytical complexity. The objective should focus on determining whether HAI retains predictive value beyond Breslow thickness alone. In addition to examining whether HAI is consistent across institutions and populations, future studies should also examine whether its weighting and calibration remain stable across institutions and patient populations. Additional studies should separately evaluate HAI thresholds versus continuous scores; external datasets may identify alternative partitions or may not support dichotomization at all [26,27].

Longitudinal sampling of inflammatory and metabolic systems will also be crucial in determining whether prospective evaluation increases the contribution of IMDI to recurrence, systemic progression, treatment response, or survival [28].

Another area for extension of the existing framework is molecular integration. Adding BRAF, NRAS, KIT, TERT-promoter, CDKN2A, PD-L1, MHC-I, MHC-II, tumor mutational burden, transcriptomic signatures, copy-number alterations, and response-to-treatment data may allow us to further elucidate whether morphological pathological characteristics, host systemic conditions, and tumor molecular programs represent distinct, interacting, or stage-specific axes of melanoma progression [29].

We believe that the Melanoma Intelligence Framework represents a potentially generalizable hierarchical organization of diverse clinical information rather than a completed clinical tool. In this cohort, this organization retained considerable information from multidimensional pathology and consistently identified the histopathological aggressiveness axis as the highest-ranked predictor of nodal metastatic propensity, while systemic inflammatory–metabolic biology provided a secondary layer of clinicobiological context.

## 5. Conclusions

Melanoma dissemination to regional lymph nodes can be approached as a biologically layered progression involving locally aggressive histopathology, systemic dysregulation of metabolism and inflammation, discordant histological and biological processes, and uncertainty regarding the modeled system. As such, these factors may be combined to form clinically relevant and analytically interpretable constructs.

This research project converted a pre-existing melanoma registry into a melanoma intelligence framework that preserved traditional pathological definitions while permitting the application of statistical modeling techniques, machine learning benchmarking, model-rule extraction, assessment of feature stability, network analysis, comparison of similarity among individual patient profiles, estimation of conformal uncertainty, and phenomapping.

One of the major findings of this study was that histopathologically confirmed metastatic involvement of regional lymph nodes was organized predominantly within one dimension representing local histopathologic aggressiveness. Additionally, the HAI was found to have the strongest and most stable predictive signal when compared with the other investigated indices, including systemic inflammatory–metabolic dysregulation alone, and retained independent association with regional lymph-node metastasis even after accounting for systemic inflammatory–metabolic dysregulation.

Furthermore, the IMDI demonstrated secondary biological contextual relevance. However, although the BHDS produced exploratory and hypothesis-generating results in this specific dataset, it did not produce statistically significant discrimination of endpoint groups by itself.

As stated earlier, the findings presented herein should be regarded as internally evaluated exploratory signals only. Therefore, they should not be considered indications of clinical practice guidelines. Instead, the described framework is intended to remain complementary to current practices in sentinel lymph-node biopsy, pathology-based staging, molecular diagnostic testing, interdisciplinary review of individual patient cases, and clinical decision making. More specifically, it may provide increased transparency, enhanced biological context, and auditability of melanoma risk modeling.

## Figures and Tables

**Table 1:** Analytical domains retained from the malignant melanoma registry. Variables were organized into biologically and clinically coherent domains to preserve interpretability and avoid treating the registry as an undifferentiated high-dimensional predictor set.

Domain	No. of Variables	Variables
Demographic	3 (7.7%)	Age, sex, and urban/rural background
Anatomical	5 (12.8%)	Face, scalp, cervical region, trunk, and limbs
Operative	6 (15.4%)	General anesthesia, alternative anesthesia, primary closure, local flap, locoregional flap, and graft reconstruction
Histopathological	8 (20.5%)	Clark level, Breslow thickness, satellitosis/in-transit metastasis, melanoma subtype, vertical growth phase (VGP), ulceration, mitoses/mm^2^, and lymphovascular invasion (LVI)
Nodal	4 (10.3%)	Lymphoscintigraphy, nodal-basin localization, palpable adenopathy, and histopathologically confirmed lymph-node metastasis (LNM)
Comorbidity	1 (2.6%)	Comorbidity profile
Systemic biological	10 (25.6%)	Ionized calcium, albumin, total calcium, corrected calcium, C-reactive protein (CRP), interleukin-6 (IL-6), vitamin D, alkaline phosphatase (ALP), glucose, and lactate dehydrogenase (LDH)
Clinical course	1 (2.6%)	Hospitalization duration
Staging	1 (2.6%)	Available disease stage
Total	39 (100%)	Analytical variables retained after removal of identifiable or non-analytical fields

**Table 2:** Principal derived melanoma intelligence constructs and their analytical roles. HAI and IMDI represent local histopathological and systemic inflammatory–metabolic axes, respectively; BHDS captures the magnitude of disagreement between these domains, while the directional phenotype preserves the direction of that discordance. NMPS, MCRS, and ESI provide model-derived representations of nodal risk, integrated clinicobiological context, and explanation stability.

Construct	Components	Construction	Scale	Analytical Role
HAI	Breslow thickness, mitotic activity, Clark level, ulceration, LVI, VGP, nodular subtype, and satellitosis/in-transit metastasis	Fold-fitted histopathological linear predictor with training-based min–max normalization	0–100; higher = greater histopathological aggressiveness	Local histopathological aggressiveness axis
IMDI	CRP, IL-6, LDH, glucose, ALP, corrected calcium, and vitamin D	Fold-fitted systemic inflammatory–metabolic linear predictor with training-based min–max normalization	0–100; higher = greater systemic dysregulation	Systemic biological axis
BHDS	HAI and IMDI ranks	|rank(HAI) − rank(IMDI)|/(n − 1)	0–1; higher = greater discordance	Magnitude of histology–biology mismatch
Directional phenotype	HAI and IMDI	Median-defined concordant and discordant states	Four categories	Direction of clinicobiological mismatch
NMPS	Model output	Model-estimated probability of histopathologically confirmed LNM	0–100	Patient-level predictive output
MCRS	HAI, IMDI, BHDS, age, anatomical site, and comorbidity	Integrated clinicobiological representation	Continuous/0–100	Multidomain risk representation
ESI	Bootstrap feature rankings	Overlap among the highest-ranked explanatory variables across bootstrap-resampled models	0–1; higher = greater stability	Explanation reproducibility

**Table 3:** HAI exceeded Breslow thickness alone by ΔAUC = +0.037 (95% CI: +0.008 to +0.066; *p* = 0.014), whereas retaining all eight histopathological variables separately produced only a small additional increase in AUC over HAI (ΔAUC = +0.012; 95% CI: −0.005 to +0.029).

Predictor	ROC AUC	95% CI	Endpoint Comparison
HAI	0.889	0.814–0.948	Median: 67.8 vs. 45.3; *p* < 0.001
Breslow thickness	0.852	0.758–0.921	Median: 5.85 vs. 1.80 mm; *p* < 0.001
Mitotic activity	0.831	0.733–0.907	Median: 14 vs. 3 mitoses/mm^2^; *p* < 0.001
Ulceration	0.791	0.681–0.876	19/22 vs. 20/50; OR: 9.5; *p* < 0.001
LVI	0.769	0.654–0.862	10/22 vs. 3/50; OR: 13.1; *p* < 0.001
Nodular subtype	0.741	0.624–0.838	15/22 vs. 12/50; OR: 6.8; *p* < 0.001
Clark level	0.718	0.598–0.818	Greater invasion depth in node-positive disease
VGP	0.598	0.478–0.710	Limited endpoint separation
Satellitosis/in-transit metastasis	0.583	0.462–0.697	4/22 vs. 3/50; OR: 3.5; *p* = 0.190

**Table 4:** Directional HAI–IMDI phenotype was associated with nodal status (Fisher exact *p* < 0.001), despite the absence of discrimination by continuous BHDS.

Phenotype	Patients, n (%)	Node-Positive, n/N (%)
Low-risk concordant	18 (25.0%)	0/18 (0%)
Histology-dominant	31 (43.1%)	16/31 (51.6%)
Biology-dominant	14 (19.4%)	2/14 (14.3%)
High-risk concordant	9 (12.5%)	4/9 (44.4%)

**Table 5:** Leave-one-out cross-validated performance of models predicting histopathologically confirmed LNM. HAI-centered models and Random Forest showed the strongest out-of-fold performance, whereas increasing model complexity did not produce a consistent gain across discrimination, probabilistic accuracy, and classification performance.

Model/Method	ROC AUC	PR-AUC	Brier Score	Balanced Accuracy	F1 Score	MCC
HAI-only penalized logistic	0.889	0.706	0.137	0.845	0.755	0.641
IMDI-only penalized logistic	0.583	0.405	0.244	0.623	0.490	0.234
HAI + IMDI + BHDS logistic	0.879	0.701	0.143	0.776	0.680	0.522
Integrated penalized logistic	0.839	0.643	0.157	0.796	0.708	0.568
Integrated elastic-net logistic	0.859	0.658	0.149	0.796	0.708	0.568
Random forest	0.881	0.708	0.136	0.857	0.764	0.661
ExtraTrees	0.835	0.704	0.190	0.751	0.652	0.490
Gradient boosting	0.805	0.499	0.153	0.630	0.489	0.257
AdaBoost	0.749	0.624	0.140	0.857	0.764	0.661
Shallow classification tree	0.775	0.537	0.171	0.857	0.764	0.661
SVM-RBF	0.814	0.602	0.164	0.700	0.579	0.443
K-nearest neighbors	0.796	0.707	0.164	0.665	0.514	0.394
Gaussian Naïve Bayes	0.733	0.551	0.620	0.515	0.465	0.043

**Table 6:** HAI accounted for the largest SHAP contribution and ranked first in every bootstrap iteration, whereas the ordering of secondary explanatory variables was less stable. The top-three ESI was 0.604. * Palpable adenopathy appeared among the five highest-ranked features in 78% of bootstrap resamples.

Feature	Mean Absolute SHAP	Relative Contribution	Top-Three Bootstrap Frequency	Median Bootstrap Rank (IQR)
HAI	0.184	35.8%	100%	1.0 (1.0–1.0)
BHDS	0.092	17.9%	52%	3.5 (2.0–5.0)
Age	0.071	13.8%	60%	4.0 (3.0–5.0)
IMDI	0.063	12.3%	74%	3.0 (2.0–4.0)
Palpable adenopathy	0.048	9.4%	— *	5.0 (4.0–6.0)
Anatomical site	0.035	6.8%	—	—
Comorbidity	0.021	4.0%	—	—

## Data Availability

Available upon reasonable request from the corresponding authors.
